# Motivations behind complementary and alternative medicine use in patients with Crohn’s disease and ulcerative colitis

**DOI:** 10.1093/jcag/gwae020

**Published:** 2024-07-31

**Authors:** Natasha Klemm, Roberto Trasolini, Brian Bressler, Gregory Rosenfeld, Gina Almasan, Yvette Leung

**Affiliations:** Department of Gastroenterology, University of British Columbia, Vancouver, BC V6B0C3, Canada; Department of Gastroenterology, University of British Columbia, Vancouver, BC V6B0C3, Canada; Department of Gastroenterology, University of British Columbia, Vancouver, BC V6B0C3, Canada; IBD Centre of BC, Vancouver, BC V6Z 2L2, Canada; Department of Gastroenterology, University of British Columbia, Vancouver, BC V6B0C3, Canada; IBD Centre of BC, Vancouver, BC V6Z 2L2, Canada; IBD Centre of BC, Vancouver, BC V6Z 2L2, Canada; Department of Gastroenterology, University of British Columbia, Vancouver, BC V6B0C3, Canada; IBD Centre of BC, Vancouver, BC V6Z 2L2, Canada

## Abstract

**Background:**

Complementary and alternative medicine (CAM) use is common in inflammatory bowel disease (IBD) patients and impacts compliance with conventional treatment. Gastroenterologists should understand the motivational factors of CAM use—factors that *push* patients away from standard therapy or *pull* towards CAM. Our study describes the motivations behind CAM use for IBD and evaluates differences between Crohn’s disease (CD) and ulcerative colitis (UC) patients.

**Methods:**

Retrospective cohort survey of patients over 18 years old with IBD, evaluated by gastroenterologists at a tertiary care referral centre from January 1 to December 31, 2019. Only patients who reported CAM use were included. Chi-square and independent *t*-tests were performed and *P*-value <0.05 was significant.

**Results:**

Of the 230 completed surveys, 193 reported CAM use (CD: 57.5% vs UC: 42.5%). Demographics, disease duration, and hospitalizations were similar, but CD patients had lower SIBDQ scores (CD: 48.1 vs UC: 53.5, *P* < 0.001). Both groups were largely influenced by their social network to use CAM (CD: 33% vs UC: 31.3%) and did not feel well informed about CAM (87.4%). CD and UC patients had similar push and pull factors. Push factors included lack of improvement (39%) and side effects (20%) with conventional treatment. Pull factors included the desire for a holistic approach (21%) and to improve mood (35%). UC patients wanted a natural approach to treat their IBD, which nearly reached significance (*P* = 0.049). Most patients hoped fatigue 62.7%, and diarrhoea 61.7% would improve with CAM, but more CD patients wanted to improve their appetite (*P* = 0.043).

**Conclusion:**

Despite differences in QoL, push and pull motivations for CAM use did not differ between CD and UC patients. Most users do not feel well informed of CAM and ongoing dialogue is important for patient-centred care.

## Graphical Abstract

**Graphical Abstract:**
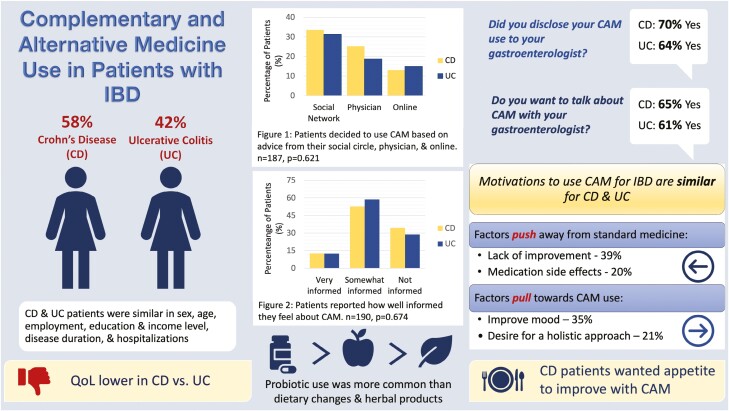

## Introduction

Inflammatory bowel disease (IBD) is an immune-mediated, chronic disease with a relapsing and remitting pattern characterized by abdominal pain, diarrhoea, and rectal bleeding. It may negatively impact quality of life (QoL) and require hospitalization and surgery.^1^ While treatment targets have evolved and newer advanced therapies are approved, patients focus on symptom management and improvement in QoL.^1^

The WHO defines complementary and alternative medicine (CAM) as “…a broad set of health care practices that are not part of a country’s own tradition nor part of conventional medicine, and are not fully integrated into the dominant healthcare system.”^2^ CAM is especially prevalent in patients with chronic disease, but given the variety of therapies and practices that are considered CAM, it is challenging to evaluate the motivations behind its use.^3,4^

Canada has one of the highest rates of CAM use, 21%–56%^5–7^ and British Columbia, has increasingly positive attitudes toward natural therapies compared to other provinces.^8^ Globally, 40%–50% of IBD patients use CAM and 18%–33% use CAM specifically for IBD.^5,7,9,10^ Predictors of CAM use in the general population include female gender, higher income, and urban dwelling.^7,11^ Initial enquiries identified various motivations behind CAM use, which are characterized as push or pull factors in this current study. Push & pull factors are an extension of Lee’s theory of physician migration and have been applied to physician, nurse, and medical trainee career choice, as well as patient healthcare utilization.^12,13^ Broadly, negative forces *push* patients away from their current situation or treatment and positive forces *pull* towards a new destination or treatment.^14^ Portela et al. were the first to characterize push and pull factors involved in CAM use by patients with IBD.^11^*Push factors*, which include side effects or dissatisfaction with standard care, push patients away from conventional treatment; whereas *pull factors*, such as the desire to have a holistic or natural approach, pull patients towards CAM.^11^ Earlier literature evaluates these factors using a broad characterization of CAM and not for IBD specifically.

The literature on specific CAM therapies is sparse, creating issues around safety and efficacy. Similarly, CAM may interact or impact compliance with standard treatment.^5,11,15^ It is imperative that gastroenterologists understand the motivations behind CAM use for IBD as addressing CAM use is integral to patient-centred care and improves outcomes.^16^ The primary aim of this study is to identify the push and pull factors that drive patients to use CAM for IBD.

## Materials and methods

### Study design

This survey-based, retrospective cohort study was conducted at the Pacific Gastroenterology Associates tertiary care, referral, outpatient clinic located in Vancouver, British Columbia and included patients with a diagnosis of IBD that were seen by a gastroenterologist between January 1, 2019 to December 31, 2019. The patients had consented to be contacted via email for future research.

Patients who met the inclusion criteria were notified by email with a detailed description of the study and a link to the survey. Prior to accessing the survey, the description clearly stated that completion of the survey provided consent to have their responses used for the study. Data from all attempted surveys was stored and electronically extracted from Qualtrics, a secure survey tool available for research through the University of British Columbia. Quality of Life (QoL) was assessed using the Short IBD Questionnaire (SIBDQ), a licensed, 10 question survey validated in both UC and CD patients that scores on bowel, systemic, social, and emotional domains.^17^ Each question contains a 7-point scale and a cut-off of <50 was used to define poor QoL. Ethics approval was obtained through the University of British Columbia Research Ethics Board.

### Inclusion/exclusion criteria

Patients met inclusion criteria if they were 18 years or older, diagnosed with IBD, and evaluated at the Pacific Gastroenterology Associates tertiary care clinic between January 1, 2019 and December 31, 2019. Patients had provided consent for email correspondence for research purposes. Due to delays in study and license approval during the COVID pandemic, the survey was sent to patients in August 2022. Patients were excluded if they did not meet the above criteria and if they did not return the survey or returned an incomplete survey. A completed survey required 70% completion of the questions, which included the completed SIBDQ and demographics.

### Data extraction and definitions

Patients meeting the inclusion criteria had the following demographic data collected in the survey: age, sex, education, employment, income, disease duration, current medication prescribed for IBD, surgical history, IBD-related hospitalizations and SIBDQ score. Participants were asked if they were currently taking medication for their IBD, which included steroids, 5-ASA products, azathioprine, methotrexate, infliximab, adalimumab, vedolizumab, ustekinumab, and tofacitinib. Participants chose predefined survey answers with a free text option if their answer were not available.

Questions about use of CAM were drawn from a national health survey on the use of Complementary and Alternative Medicine by Canadians.^8^ CAM was characterized as dietary changes, natural supplements/herbal products, mental health services, and alternative healthcare services. Specific therapies, including herbal products, were chosen for the survey-based on previous reviews on the efficacy and potential mechanism of individual CAM therapies in IBD.^15,18,19^ Patients were given the option to report additional types of CAM used for IBD, ensuring patients who used non-listed CAM therapies were included in the study. CAM use was determined by asking participants ‘Have you ever used the following therapies for your IBD specifically?’ and the available answers were gluten-free diet, anti-inflammatory diet, other dietary changes, probiotics, curcumin, *Andrographis paniculata* (Indian Echinacea), Boswellia serrate (Indian frankincense), *Artemesia absinthium* (wormwood), Cannabis, other herbal formulas, Cognitive Behavioural Therapy, Acupuncture and other. The “other” option provided a free text box to input CAM products or services that were not specifically listed. For each chosen answer, participants were asked to report the IBD symptom(s) that the CAM product or service was used for. The survey asked about monthly expenditure on CAM, motivations for use, source of information, degree of CAM knowledge, and disclosure to their gastroenterologist.

### Data analysis

Data was extracted from Qualtrics and analyzed using SPSS 27. Participants were separated and compared based on their IBD type, CD or UC. Continuous variables were described using median, mean, standard deviation, and minimum/maximum values when applicable. Categorical variables were described using absolute frequencies and analyzed using cross tabulations and chi-square test. A significance level of 5% was used for all tests.

## Results

### Excluded patients

The survey was sent to 910 participants; 11 emails bounced and there were 2 duplicate emails. 293 surveys were started and 253 surveys were submitted. Of these, 230 surveys were completed and 193 patients reported CAM use.

### Study demographics

Of the CAM users, 57.5% (111/193) had CD and 42.5% (82/193) had UC. There was no difference in the mean age, sex, education level, employment, income, disease duration, and hospitalizations between the phenotypes (Table 1). CD patients scored lower on the SIBDQ (CD:48.1 vs UC:53.5 *P* < 0.001), had a greater number of surgeries (CD: 73, 65%, vs UC: 11, 13%, *P* < 0.001), and increased use of immunosuppression (CD: 9.9% vs UC: 2.4%, *P* = 0.041) and anti-TNF biologics (CD: 35.1% vs UC 15.9%, *P* < 0.001) (Table 2). UC patients had a significantly higher rate of 5-ASA derivatives (CD: 9.9% vs UC 37.8%, *P* < 0.001) (Table 2).

**Table 1.** Study demographics of IBD patients that use CAM.

Gender, *n* = 193 (%)	
Male	79 (40.9)
Female	113 (58.5)
Prefer not to say	1 (0.9)
Age, mean, *n* = 193	46.8 ± 15.8
Employment status, *n* = 192 (%)	
Full time	101 (52.6)
Part time	16 (10.0)
Student	6 (3.0)
Not working	43 (22.4)
Other	26 (13.5)
Education *n* = 192 (%)	
No post-secondary	20 (10.4)
Some post-secondary	71 (37.0)
University	101 (52.6)
Income *n* = 189 (%)	
<40,000	45 (23.8)
40-59 000	35 (18.5)
60-79 000	32 (16.9)
>80 000	77 (40.7)

**Table 2.** Disease history of IBD patients that use CAM.

Disease duration *n* = 193 (%)	
<12 months	1 (1.2)
1-5 years	18 (9.0)
6-10 years	45 (23.3)
11-20 years	71 (36.8)
>20 years	58 (30.1)
Medical therapy *n* = 193 (%)	
Steroids (*n* = 191)	9 (5.0)
5-ASA derivatives	42 (21.8)
Azathioprine/methotrexate	9 (5.0)
Anti-TNF	52 (26.9)
Newer agents	79 (40.9)
Combination	6 (3.0)
No advanced therapy	50 (25.9)
Hospitalizations *n* = 158 (%)	
None	51 (32.3)
Within past month	1 (1.0)
Within past 12 months	4 (3.0)
Within past 5 years	36 (22.8)
Within past 10 years	66 (41.8)
Surgeries *n* = 193 (%)	
None	109 (56.5)
1	63 (32.6)
2	9 (4.6)
3 or more	12 (10.8)

### Types of CAM use

Overall, probiotics and dietary changes were the most commonly used CAM by IBD patients (Table 3), however, there was no statistical difference between the use of a gluten-free diet, anti-inflammatory diet, probiotics, curcumin, herbal products, cannabis, cognitive behavioural therapy, and acupuncture between the CD and UC groups (Table 3). There was a numerical trend towards increased Cannabis use by CD compared to UC patients (CD: 42, 37.8% vs UC:21 25.6%, *P* = 0.073). Herbal products were used the least (Table 3).

**Table 3.** Characteristics of CAM use.

	CD	UC	*P*-value
*Have you ever used the following therapies for your IBD? n = 193 (%)*			
Gluten-free diet	44 (39.6)	36 (43.9)	0.552
Anti-inflammatory diet	51 (45.9)	34 (41.5)	0.535
Other diet	53 (47.7)	37 (45.1)	0.718
Probiotics	70 (63.1)	57 (69.5)	0.35
Curcumin	21 (18.9)	24 (29.3)	0.093
Andrographis paniculata	0	0	
Boswellia serrate	4 (3.6)	3 (3.7)	0.984
Artemisia absinthium	5 (4.5)	0	0.052
Cannabis	42 (37.8)	21 (25.6)	0.073
Other herbal formula	17 (15.3)	18 (22.0)	0.237
Cognitive behavioural therapy	20 (18.0)	12 (14.6)	0.532
Acupuncture	28 (25.2)	18 (22.0)	0.598
Other	25 (22.5)	16 (19.5)	0.613
*What are the reasons for using CAM? n = 193 (%)*			
Conventional medicine did not make me feel better	46 (41.4)	29 (35.4)	0.392
Conventional medicine will not cure my disease	24 (21.6)	14 (17.1)	0.432
Side effects of conventional medicine	22 (19.8)	17 (20.7)	0.876
Final hope	15 (13.5)	11 (13.4)	0.984
Fear of surgery	20 (18.0)	9 (11.0)	0.176
Want to use natural approach	17 (15.3)	22 (26.8)	0.049
Want to use holistic approach	22 (19.8)	18 (22.0)	0.718
Improve mood	41 (36.9)	26 (31.7)	0.451
Other	47 (42.3)	30 (36.6)	0.419
*What symptoms were you hoping would improve with CAM? n = 193 (%)*			
Abdominal pain	69 (62.2)	43 (52.4)	0.176
Diarrhoea	64 (57.7)	55 (67.1)	0.184
Fatigue	72 (64.9)	49 (59.8)	0.468
Appetite	28 (25.2)	11 (13.4)	0.043
Weight loss	24 (21.6)	14 (17.1)	0.432
Other	34 (30.6)	21 (25.6)	0.445

### Motivations for CAM use

**Figure 1.**
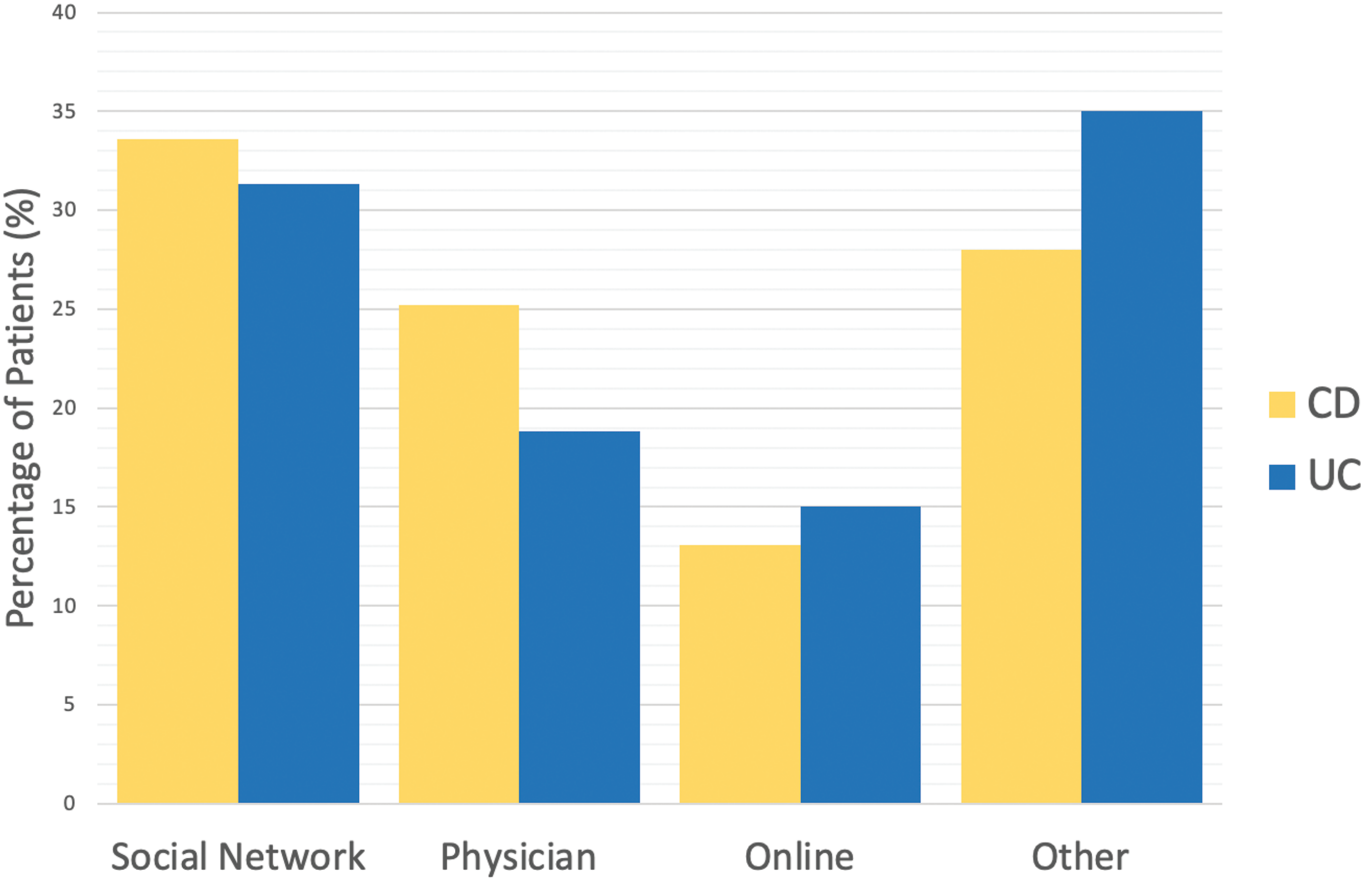
Percentage of patients that decided to use CAM based on advice from their social circle, physician, online, or other, *n* = 187, *P* = 0.621; CD = Crohn’s disease; UC = ulcerative colitis.

**Figure 2.**
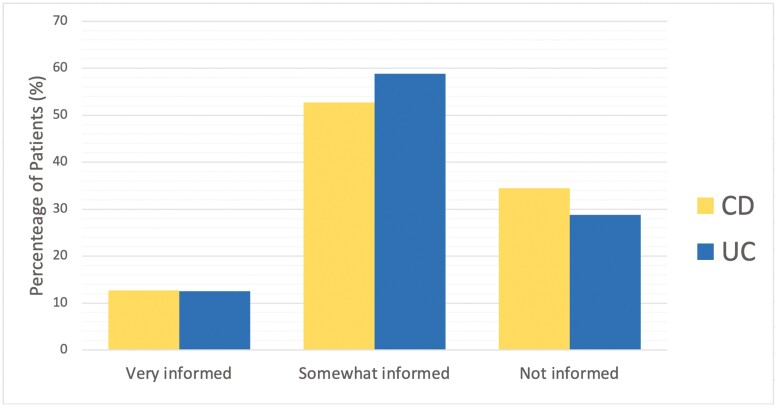
Patients reported how well informed they feel about CAM, *n* = 190, *P* = 0.674; CD = Crohn’s disease; UC = ulcerative colitis.

**Figure 3.**
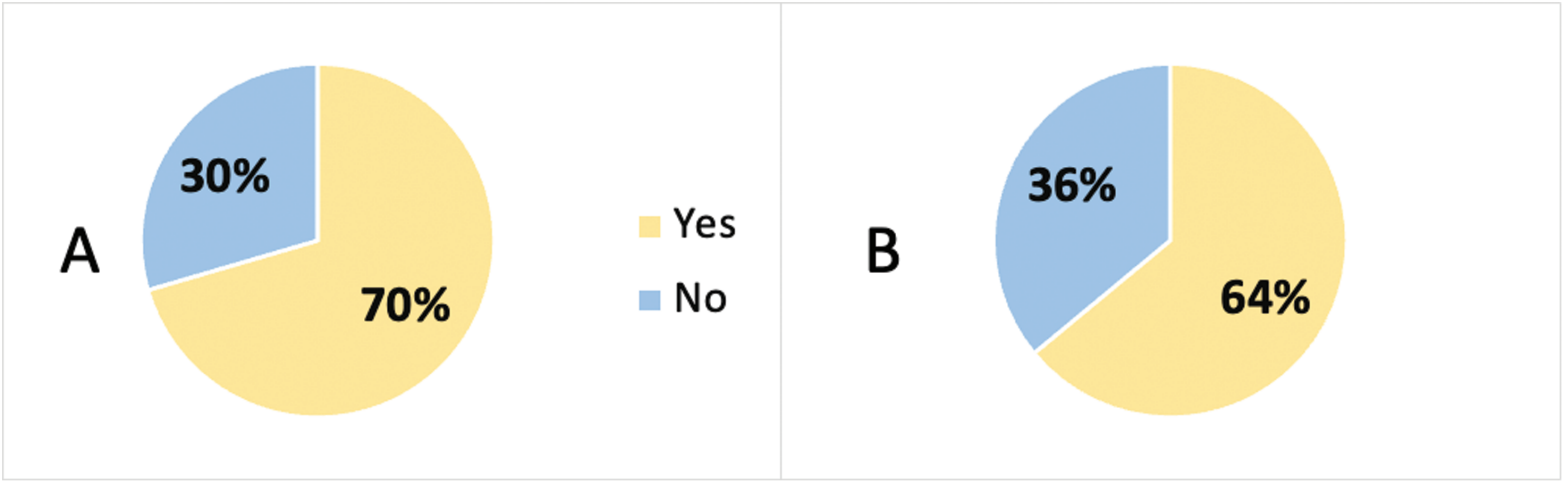
Percentage of CD and UC patients that disclosed CAM use to their gastroenterologist, *n* = 180, *P* = 0.359. (A) = Crohn’s disease; (B) = Ulcerative colitis.

**Figure 4.**
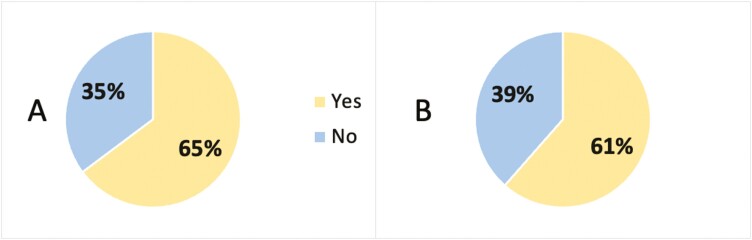
Percentage of CD and UC patients that wanted to discuss CAM with their gastroenterologist, *n* = 183, *P* = 0.654. (A) = Crohn’s disease; (B) = Ulcerative colitis.

Both groups primarily decided to use CAM based on advice from a colleague, friend, or family member (CD:33% vs UC:31.3%, *P* = 0.621, Figure 1). CAM users did not feel well informed of CAM therapies (166, 87.4%, *P* = 0.674, Figure 2), but the majority of participants disclosed their CAM use (CD:74, 70.5% vs UC:48, 64%, *P* = 0.359, Figure 3) and wanted to discuss CAM (CD: 68, 64.8% vs UC: 48, 61.5%, 0.654, Figure 4) with their gastroenterologist. CD and UC patients had similar push and pull motivational factors; although, nearing significance, more UC patients wanted a natural approach (*P* = 0.049, Table 3). Push factors included side effects, fear of surgery and that standard medical treatment did not make them feel better and would not cure their disease. Pull factors included the desire to use a holistic approach and to improve mood (Table 3). The primary symptoms that IBD patients wanted to improve with CAM were fatigue 62.7% (121/193), diarrhoea 61.7% (119/193), and abdominal pain 58.0% (112/193), but more CD patients hoped appetite (*P* = 0.043) would improve (Table 3).

## Discussion

### Motivations

Our study demonstrates that both push and pull factors are responsible for CAM use by IBD patients, to address their condition. The lack of improvement in symptoms with conventional medicine—a push factor—was the most common motivation. This is similar to earlier studies demonstrating lack of efficacy and side effects of conventional treatment as main drivers of CAM use.^5,6,11^ Improving mood, a pull factor, was the second most common motivation and patients with IBD have increased rates of psychiatric comorbidities.^20–22^

Both groups used CAM to improve abdominal pain, diarrhoea, and fatigue, similar to an earlier Canadian study, that used a broader definition of CAM.^23^ Diarrhoea and pain are expected to improve before endoscopic inflammation with treatment and may reflect suboptimal efficacy of medical treatment, or co-existing conditions. Assessment of CAM use may optimize care by identifying persistent symptoms.

### Type of CAM use

There was no significant difference in the type of CAM used between CD and UC patients. Similarly, the Manitoba IBD Cohort Study found no significant difference between the IBD phenotypes.^7^ In the present study, dietary changes and probiotics were the most common type of CAM used by both groups. Probiotics are frequently the most common CAM product used for both general health and IBD in CD and UC patients.^5,6^ However, Portela et al., found that herbal medicines were most commonly used, relating to regional variations in CAM use.^5,6^ Cannabis use occurred in 33% (63/193) of the surveyed patients. This is lower than a Canadian study showing significantly increased cannabis use by IBD patients, 74% compared to a non-IBD population 48.3%.^24^ The higher rates may reflect selection bias and voluntary nature of the survey. In our study, cannabis use was unexpectedly low despite recent legalization in Canada. A possible explanation may be general attitudes of CAM users recognizing cannabis as a recreational substance, rather than medicinal. Cannabis use should be assessed due to unknown long-term effects, safety profile, and medication interactions.

### CAM disclosure

Patients used advice from a colleague, friend, or family member 32.6% (61/187) when deciding to use CAM, but did not feel well-informed 87.4% (166/193), which is similar to previous studies.^5,11,25,26^ Our study reported 63% (122/193) of patients disclosed CAM use to their gastroenterologist, which differs from previous 54%–75% rates of non-disclosure.^25^ A prospective study of 380 IBD patients demonstrated greater compliance with conventional medicine in those that disclosed CAM use, potentially indicating greater trust with their physician.^6^ In contrast, patients seek CAM due to dissatisfaction with the patient–doctor relationship^25^ and poor physician communication.^27^ CAM use and disclosure may be reflective of the therapeutic relationship.

### Strengths

The study population is comparable to an outpatient IBD population. Patients with CD had lower QoL scores and higher anti-TNF use. Unlike previous literature, our study focused on CAM use specifically for IBD, and the motivating factors. This provides a foundation for gastroenterologists to open dialogue with patients and avoid consequences of CAM use, such as drug interactions and non-compliance.^5,11^ Additionally, our study included specific CAM products and services frequently promoted by alternative practitioners based on limited *in vitro* and *vivo* research. Gastroenterologists can become familiar with relevant literature and navigate misconceptions of efficacy, safety, and avoid dismissal of alternative therapies, as patients using CAM tend to incorporate and maintain healthy behaviour changes.^28,29^

### Limitations

A limitation of our study is the survey design and potentially polarizing topic of CAM, which is likely to attract participants with a strong interest in CAM. This may explain the lower response rate, particularly by CAM non-users. Future studies can provide the survey at the initial appointment rather than through email at a later date. Many patients had disease duration greater than a decade, and recall bias may be present. Our study asked about specific CAM products and services, which limits comparison to studies that hold broader CAM definitions. A standardized CAM questionnaire may alleviate study protocol differences, although this has not been adopted in the literature.^30^

## Conclusion

Patients with IBD are motivated to use CAM by both push and pull factors, despite having little knowledge of CAM products and services. Lack of symptom improvement with conventional therapies and mental health concerns are the most common drivers of use. By discussing CAM, gastroenterologists have an opportunity to identify persistent symptoms, optimize treatment and compliance, and improve the therapeutic relationship.

## Supplementary Material

gwae020_suppl_Supplementary_Material

## Data Availability

The data underlying this article will be shared on reasonable request to the corresponding author.
